# Focal Placenta Accreta in a Congenitally Malformed Uterus: A Case Report

**DOI:** 10.7759/cureus.47618

**Published:** 2023-10-25

**Authors:** Asma Fahad, Atif Fazari, Nahla Al Fardan, Umniyah Abu-nayla, Ayat Haseep, Noor Alabdi

**Affiliations:** 1 Obstetrics and Gynaecology, Latifa Hospital, Dubai Academic Health Corporation, Dubai, ARE; 2 Obstetrics and Gynaecology Residency Program, Dubai Academic Health Corporation, Dubai, ARE

**Keywords:** classical cesarean section, uterine septum resection, bicornuate uterus, placenta accreta, morbidly adherent placenta

## Abstract

Placenta accreta is defined as an abnormal trophoblast invasion of part or all of the placenta into the myometrium of the uterine wall. It is a well-known cause of maternal morbidity and mortality. Here, we present a unique case of focal placenta accreta due to a bicornuate uterus and a history of septum resection. We also discuss its management and outcome.

The patient underwent a classical cesarean section and reinforcement of the anterior and posterior uterine wall. The patient had a history of surgery for correction of uterine malformation, which may have resulted in an abnormal adherence of the placenta.

## Introduction

Placenta accreta, a condition characterized by an abnormal trophoblast invasion of part or the entire placenta into the myometrium of the uterine wall [1], is a significant and well-recognized contributor to maternal morbidity and mortality. Placenta accreta spectrum (PAS) disorders have emerged as a growing concern worldwide. They pose a substantial risk to maternal well-being during childbirth. Recent studies have underscored the severity of this issue, revealing an alarming 18-fold increase in maternal morbidity associated with PAS disorders [2].

In this case report, we present an exceptional instance of focal placenta accreta, distinguished by the presence of a bicornuate uterus and a history of septum resection. This report describes the management of this unique case and provides insights into its clinical outcome.

## Case presentation

A 43-year-old female presented to us at 31 weeks of gestation with a case of suspected placenta accreta. She was para 2 with a history of two prior deliveries via cesarean section. Her first pregnancy ended in a miscarriage at eight weeks. In her second pregnancy, she had an unexplained intrauterine fetal death at 36 weeks, and a live fetus was delivered in her third pregnancy. She had undergone laparoscopic surgery for uterine septum removal after her first pregnancy; however, no operative details were available to the patient.

She was referred to our facility for her high-risk pregnancy and attended at 31 weeks of gestation. A general examination showed stable vitals, and an abdominal examination showed a fundal height corresponding to gestational age.

A multidisciplinary team consisting of a high-risk obstetrician and the fetal medicine unit managed the patient. An ultrasound scan showed the following: the placenta was anterior low, lying 1.5 cm away from the os. The surface of the placenta looked smooth, with few and small lakes; the bladder wall was also smooth. The retroplacental hypoechoic area was maintained all over, and the myometrium wall was smooth and maintained. The thinnest part was about 2.4 mm. There was moderate vascularity on a color Doppler.

The patient was planned for elective delivery by classical cesarean section at 35 weeks, given the suspicion of placenta accreta. High-risk surgical consent was obtained.

Intraoperatively, a vertical skin incision was done, and the abdominal wall was opened in layers. Severe omental adhesions were encountered between the uterus and the anterior abdominal wall, which were separated using Harmonic and LigaSure.

The uterus was exteriorized and opened by a classical incision at the fundus. The fetus was in a transverse lie and was delivered weighing 1,855 g and handed over to the neonatology team.

**Figure 1:**
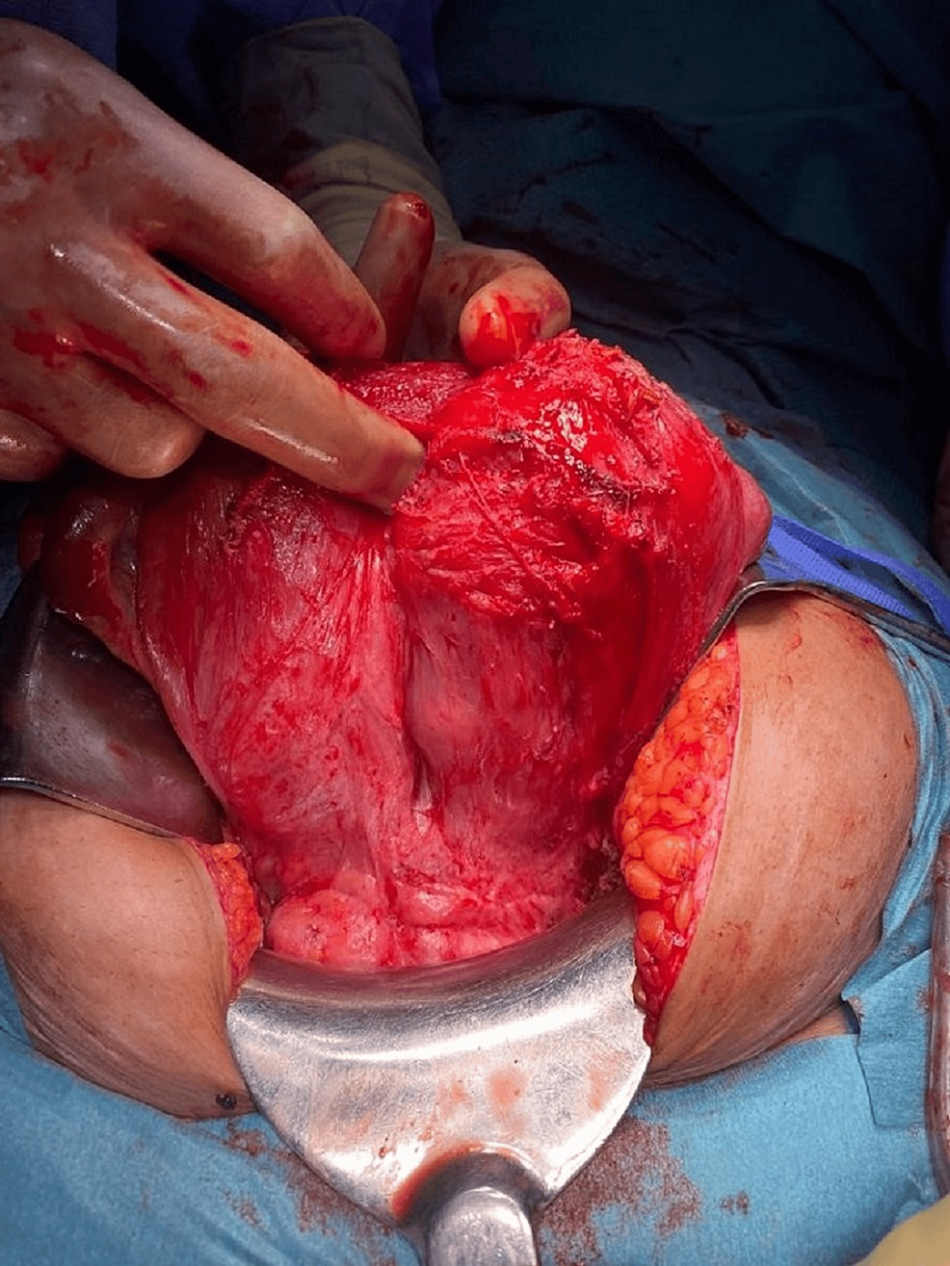
Indentation at the anterior surface of the uterus at the level of the uterine septum.

**Figure 2:**
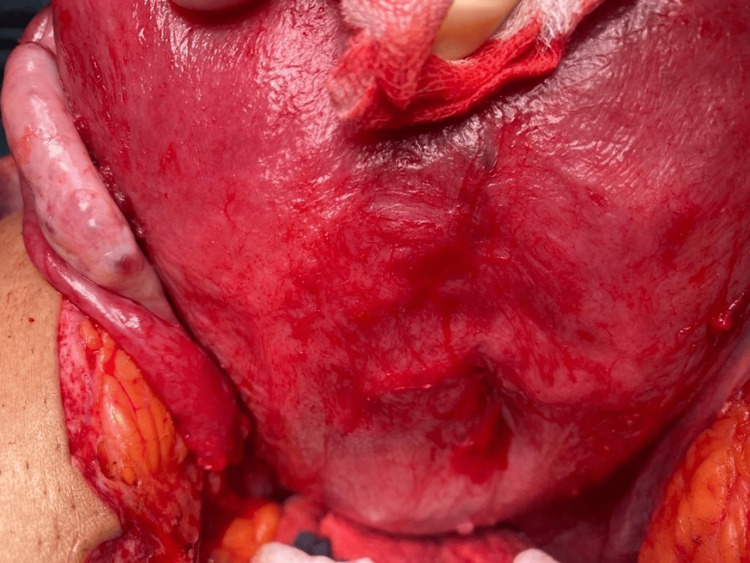
Indentation at the level of the uterine septum posteriorly.

The umbilical cord stump was ligated, and the uterus was closed using mass closure. The bladder peritoneum was separated from the uterine wall, and the lower segment was noted to be paper thin with an area of focal accreta with vertical indentation anteriorly and posteriorly (at the level of the uterine septum) (Figures 1, 2).

**Figure 3:**
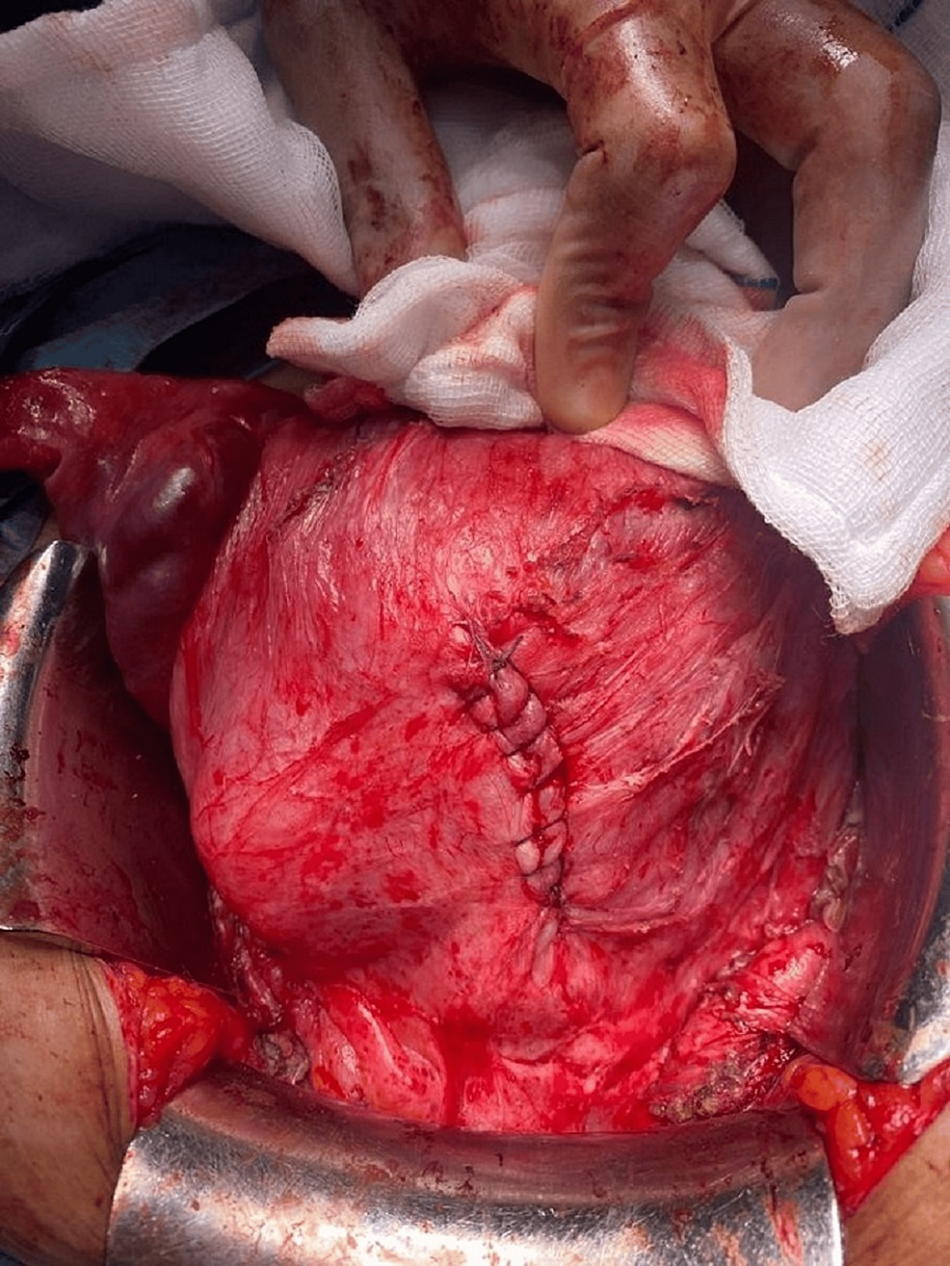
Reinforced anterior uterine wall.

The placenta was separated and delivered manually in pieces from the fundal incision. No myometrial tissue was felt anteriorly on removing the placenta. The fundal incision was closed in three layers. An incision was made in the lower segment in the thin area. The uterine septum was excised, and the cavity was reconstructed (Figure 3).

**Figure 4:**
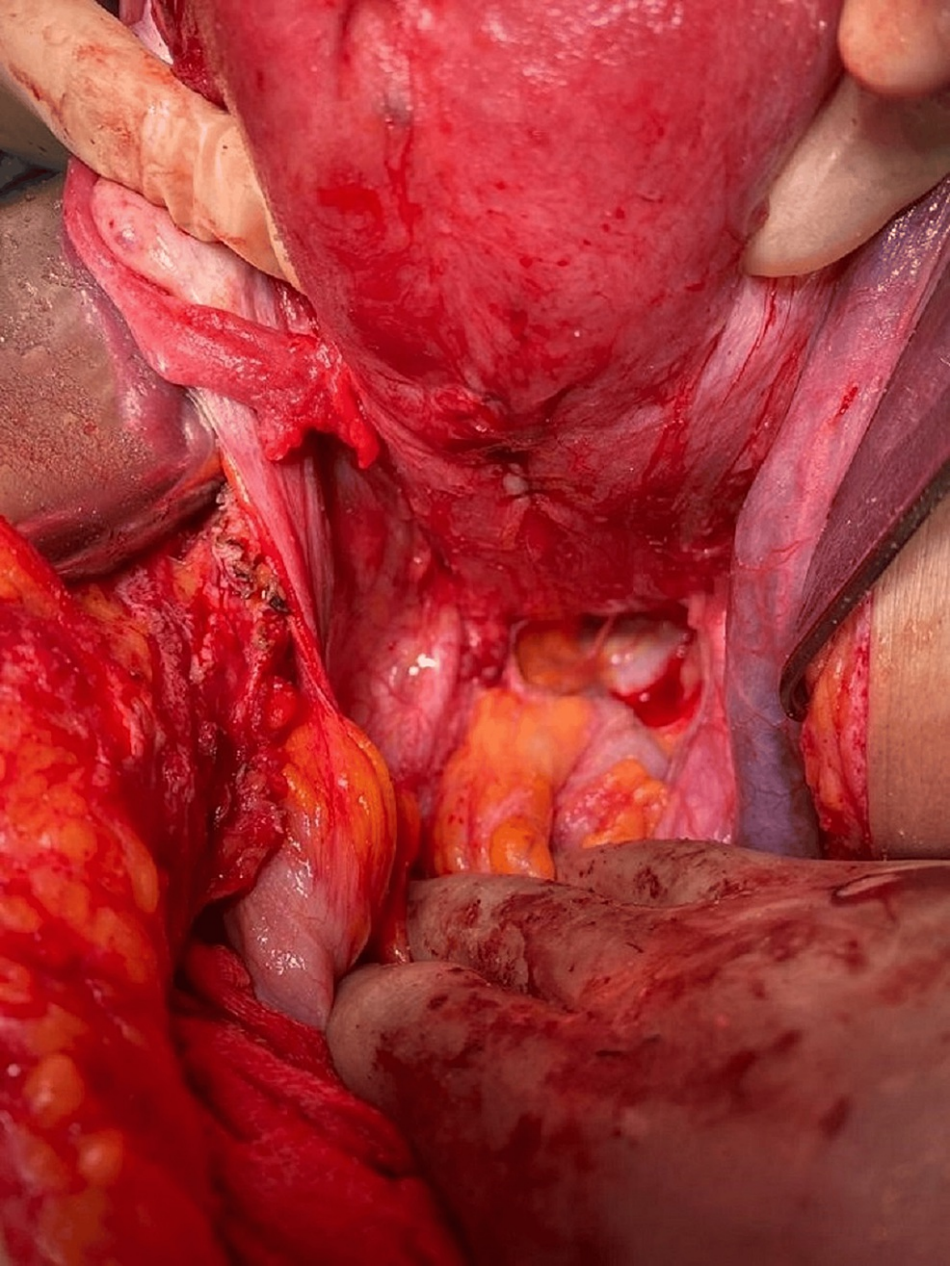
Reinforced posterior uterine wall.

Reinforcement of the lower segment was done. Posteriorly, the vertical indented area of weakness was sutured and reinforced (Figure 4).

The abdomen was closed in layers. The total estimated blood loss was 800 mL. Postoperatively, the patient recovered well and was discharged in good condition. The baby stayed in the neonatal intensive care unit for 13 days and was discharged in stable condition.

Microscopic description of the first specimen sent for histopathology is as follows: Sections showed the uterus lined by decidua pregnancy with a clear demarcation between the decidua and the myometrium. In one slice, intermediate trophoblasts were seen to infiltrate into the muscle wall. The architecture of the myometrium and its vascularity were preserved as intermediate trophoblasts and were seen as single infiltrative cells rather than forming a mass lesion. Cytological atypia was not seen, and no mitoses were identified. No chorionic villi were seen in the sections examined. There was no evidence of significant inflammation, granuloma, or neoplasia. The overall morphology was in keeping with the exaggerated placental site. Clinical correlation was recommended. It was concluded that the uterus showed an exaggerated placental site. There was no evidence of neoplasia.

Microscopic description of the second specimen sent for histopathology is as follows: There were fragments of slightly edematous smooth muscle tissue. No chorionic villi and no intermediate trophoblasts were seen. There was no evidence of significant inflammation, atypia, or malignancy. Clinical correlation was recommended. It was concluded that the uterine septum showed slightly edematous smooth muscle tissue.

## Discussion

The term morbidly adherent placenta is an umbrella term often used to describe a spectrum of pathologic placental invasion into the uterine wall. The depth of invasion into the uterine wall allows for grading of abnormal placental attachment. Placenta accreta is an invasion into decidua basalis, increta is an invasion into the myometrium, and percreta is when placental chorionic villi fully penetrate the myometrial wall, breaching the serosa and invading surrounding structures (e.g., bladder, broad ligament, sigmoid colon) [3].

Placenta accreta is an increasingly prevalent and potentially devastating obstetric complication. Its initial suspicion often arises after abnormal findings are detected via obstetric ultrasound in asymptomatic patients. These atypical ultrasound findings encompass large, irregular placental lacunae, often presenting with a “moth-eaten” appearance, typically exhibiting turbulent internal flow [4].

Early diagnosis is essential for appropriate counseling and subsequent management, and an ultrasound examination is the reference standard for diagnosis [5]. Although patients with placenta previa may experience initial bleeding, the primary clinical manifestation generally presents as a profuse, life-threatening hemorrhage during attempted manual placental separation [6].

In cases of typical placentation, trophoblast invasion is constrained to the spongiosus layer of the decidua. Several theories have attempted to elucidate the etiology of placenta accreta. A prominent hypothesis suggests that, in individuals with a history of uterine surgeries, the spongiosus layer of the decidualized endometrium may be absent, thus eliminating the conventional halt signal [7]. Noteworthy risk factors for clinically significant placenta accreta include placenta previa, prior cesarean delivery, previous uterine instrumentation, multiparity, and advanced maternal age [8].

The revised American Fertility Society classification defines a bicornuate uterus as a double uterus with a single cervix resulting from incomplete lateral fusion of the two Müllerian ducts. The bicornuate uterus can be categorized as partial or complete based on the extent of division in the uterine corpus [9]. It can contribute to reduced muscle tissue, abnormal blood flow, and cervical incompetence, potentially leading to infertility, abortion, preterm delivery, intrauterine growth restriction, and malpresentation [10].

Surgical interventions, such as the Strassman metroplasty, which can be performed abdominally or laparoscopically, have demonstrated the capacity to enhance uterine morphology, increase cavity volume, reduce intrauterine pressure, and augment blood flow to the endometrium and muscle [11]. However, it is noteworthy that placenta previa and accreta can still occur in a bicornuate uterus because of the presence of scar tissue resulting from prior metroplasty and curettage procedures. In a case report by Zhang et al., a patient who had undergone laparoscopic Strassman metroplasty for a complete bicornuate uterus experienced significant bleeding during surgical delivery at 37 weeks of gestation, attributed to placenta previa [12].

## Conclusions

In this case, the patient presented with a known history of a bicornuate uterus and had undergone surgical resection in her home country. Subsequent scans performed at our healthcare facility led to the diagnosis of placenta accreta. These combined factors, along with the patient’s medical history, significantly influenced the course of her care and led to our decision to proceed with a classical cesarean section. During the surgical procedure, we successfully resected and reconstructed the uterine septum, achieving a positive outcome for the patient.
